# Identification of specific and semi-specific SIRT inhibitors through computer-aided studies

**DOI:** 10.18632/aging.100387

**Published:** 2011-09-19

**Authors:** Antonello Mai

**Affiliations:** Istituto Pasteur-Fondazione Cenci Bolognetti, Dipartimento di Chimica e Tecnologie del Farmaco, Sapienza Università di Roma, Roma, Italy

NAD^+^-dependent lysine deacetylases (sirtuins, SIRT1-7) have emerged as potential therapeutic targets for treatment of human illnesses such as cancer, metabolic, cardiovascular and neurodegenerative diseases. Sirtuins possess deacetylase and/or mono-ADP-ribosyltransferase activity, and this activity is directed to histone as well as non-histone targets involved in transcription, metabolism, and energy homeostasis [1,2]. SIRT1, having in cells a nuclear localization, has been widely recognized to play a multifaceted, protective role in aging, metabolism, and neurodegeneration [3]. In cancer, the role of SIRT1 is highly debated. Among SIRT1/2 inhibitors, sirtinol induced senescence-like growth arrest in human breast cancer MCF-7 cells and lung cancer H1299 cells [4] and inhibited cell growth in prostate cancer [5]; cambinol induced apoptosis in BCL6-expressing Burkitt lymphoma cells [6]; salermide was well tolerated by mice at concentrations up to 100 μM and prompted tumor-specific apoptosis in a wide range of human cancer cell lines [7]; MC2141 displayed high antiproliferative activity against Raji, DLD1, and HeLa cells [8], and tenovins, identified via a yeast genetic screen for p53 activators, decreased tumor growth in vivo as single agents at low micromolar concentrations [9]. On the other hand, in some contexts SIRT1 seems to have a protective role in cancer, in particular in colon cancer. SIRT2 is a cytoplasm enzyme mainly known as α-tubulin deacetylase, highly involved in cell cycle regulation. SIRT2 crucially regulates the functions in the mitotic checkpoint elicited by mitotic stress, as well as cell death in response to DNA damage-inducing stress [10]. In addition, SIRT2 influences adipocyte differentiation by deacetylation of FOXO proteins. Despite early evidences suggested SIRT1 as the main sirtuin target to inhibit for obtaining anticancer properties, recently SIRT2 down-regulation has been described to lead to apoptosis without cell cycle arrest in HeLa cells [11]. SIRT3-5 are mithocondrial deacetylases or ADP-ribosylases (SIRT4), and control adaptive thermogenesis (SIRT3), aging (SIRT3), insulin secretion (SIRT4), and ammonia detoxification (SIRT5) [1]. Finally, SIRT6 and SIRT7 are two nuclear and nucleolar enzymes, the first involved in the control of genomic DNA stability and DNA repair as well as glucose homeostasis, the latter exerting antiapoptotic properties [1].

In this month issue of *AGING*, Schlicker et al. described the identification of specific and semi-specific SIRT inhibitors through virtual screening performed by docking 1990 structurally different compounds into the peptide binding pockets of crystal structures of SIRT2, −3, −5, and −6. To avoid to select compounds blocking the NAD^+^ binding site, that is common to all the sirtuins and could highlight non isoform-specific compounds, the four SIRTs/NAD^+^ complexes have been used. For each docking run, the 10 top-ranking compounds have been selected and tested against SIRT2, −3, −5, and −6. Among the 20 compounds found active, 14 were selective for SIRT2, and 6 were able to inhibit, in addition to SIRT2, one (3 compounds) or two (2 compounds) or all (1 compound) of the other tested sirtuins. Interestingly, some compounds behaved as SIRT5 and/or SIRT6 activators. Two selected SIRT2-specific inhibitors, CSC8 and CSC13, bearing a steroid scaffold were selected for further studies. Dose-response curves gave IC_50_ values against SIRT2 of 4.8 (CSC8) and 9.7 (CSC13) μM. Tested against SIRT1, the two compounds showed weak inhibition at 100 μM. In functional tests in HEK cells, CSC13 increased the acetyl-α-tubulin level at 100 μM, thus confirming its SIRT2 inhibition at a cellular level. Such compound in particular seems to be interesting for further development, since carrying a 2-phenylpyrimidine moiety fused to the tetracyclic gonane structure, it is not predicted to interact with nuclear receptors, and thus it should be devoid of steroid receptor-mediated side effects.
